# Speciation Variation and Comprehensive Risk Assessment of Metal(loid)s in Surface Sediments of Intertidal Zones

**DOI:** 10.3390/ijerph15102125

**Published:** 2018-09-27

**Authors:** Baocui Liang, Xiao Qian, Shitao Peng, Xinhui Liu, Lili Bai, Baoshan Cui, Junhong Bai

**Affiliations:** 1 State Key Laboratory of Water Environment Simulation, School of Environment, Beijing Normal University, Beijing 100875, China; girllbc@163.com (B.L.); qianwa1990@163.com (X.Q.); 18800181681@139.com (L.B.); cuibs@bnu.edu.cn (B.C.); junhongbai@163.com (J.B.); 2 Tianjin Research Institute for Water Transport Engineering, Centre for Environmental Science and Technology, Tianjin 300456, China; pengshitaotj@163.com

**Keywords:** heavy metal, intertidal zone, risk assessment, source, speciation

## Abstract

Speciation variation and comprehensive risk assessment of metal(loid)s (As, Cd, Cr, Cu, Mn, Ni, Pb and Zn) were investigated in surface sediments from the intertidal zones of the Yellow River Delta, China. Results showed that only the concentrations of As, Cd and Pb were significantly different between April and September (*p* < 0.01). In April, the residual fraction (F4) was predominant for As, Cr, Cu, Ni and Zn. However, the exchangeable and carbonate-associated fraction (F1) was dominant for Cd averaging 49.14% indicating a high environmental risk. In September, the F4 fraction was predominant and the F1 fraction was very low for most metal(loid)s except Cd and Mn. The geo-accumulation index (*I*_geo_), the F1 fraction and potential ecological risk index (PERI) of most metal(loid)s were relatively low in surface sediments for both seasons. But Pb, As and Ni were between the threshold effect level (TEL)and the probable effect level (PEL) for 66.67%, 83.33% and 91.67% in April and As and Ni were between TEL and PEL for 41.67% and 91.67%, which indicated that the concentration of them was likely to occasionally exhibit adverse effects on the ecosystem. Although the *I*_geo_, the F1 fraction or PERI of Cd in both seasons was higher at some sites, the results of sediment quality guidelines (SQGs) indicated that the biological effects of Cd were rarely observed in the studied area.

## 1. Introduction

Heavy metals and As are one of the serious pollutants in the natural environment due to their toxicity, abundance and persistence [1]. The sources of metal(loid)s in the sediments can generally be categorized into two kinds: natural source and anthropogenic source [2]. Anthropogenic activities such as reclamation, fish culture and the discharge of industrial effluents contribute mostly to the metal(loid) pollution in aquatic environments [3,4]. What’s more, heavy metals and As can be taken up and accumulated through food webs directly influencing marine organisms and human populations [5]. Therefore, the environmental risk assessment of heavy metal or As pollution is of great significance to environment and human health.

Sediments have been recognized as sensitive indicators for monitoring contaminants [6,7]. The distribution of heavy metals and As in sediments is an important research because it not only reflects the quality of coastal water but also provides useful information on the transportation and fate of pollutants [8]. Heavy metals and As in sediments exist in mobile and immobile forms. Bioavailable species of heavy metals and As have greater environmental risks, whereas the latter have lower risks to be released into the pore water and further accumulated by plants and so on [9,10]. Thus, it is important to investigate the heavy metal or As speciation in sediments, which contributes to assess the environmental risk of heavy metal or As pollution scientifically. Season and ecological type could influence the heavy metal or As distribution in aquatic ecosystems [11,12]. Consequently, it is necessary to evaluate the spatial-temporal variation of heavy metals and As in order to monitor the environment quality variation and contamination trend. Spatial-temporal distributions of heavy metals or As in different aquatic ecosystems have been documented in previous studies [1,13,14]. However, few studies have systematically investigated the spatial distribution and seasonal variation of heavy metals and As in sediments of the intertidal zones.

Only the appropriate risk assessment method can objectively reflect the degree of total metal pollution and the potential ecological risks of heavy metals and As in sediments. The geo-accumulation index (*I*_geo_) can intuitively reflect the degree of total metal pollution and accumulation but the chemical speciation and geographical spatial heterogeneity factors for different heavy metals or As are ignored. Although the potential ecological risk index (PERI) considers both the total concentration and the toxicity of heavy metals or As, it involves a high level of subjectivity and the chemical speciation is neglected [15]. Risk assessment code (RAC) provides a better interpretation of the relationship between the bioavailable fraction and mineral mobility [5]. Sediment quality guidelines (SQGs) provide an effective tool to assess the adverse biological effects of heavy metals in sediments [16,17]. Previous studies mainly adopted several risk assessment methods mentioned above for heavy metals in sediments, which could make evaluation results relatively one-sided. In order to make a more accurate assessment of heavy metal or As pollution in sediments, the complementary approaches are required. Up until now, there have been no studies to comprehensively assess the environmental risk of heavy metal or As pollution in sediments from the intertidal zones of the Yellow River Delta.

As special zones, the characteristics of intertidal zones such as tides and hydrodynamics can vary along with time and space [18]. What’s more, intertidal zones that closely link marine and terrestrial ecosystems have a great impact on the occurrence and fate of heavy metals. Therefore, the present study aims: (1) to identify the sources of As, Cd, Cr, Cu, Mn, Ni, Pb and Zn in sediments of the intertidal zones; and (2) to analyze temporal and spatial variation of heavy metals and As speciation; and (3) to assess the concentration and potential ecological risk of heavy metals and As in sediments of the intertidal zones.

## 2. Materials and Methods

### 2.1. Study Area and Sample Collection

**Figure 1:**
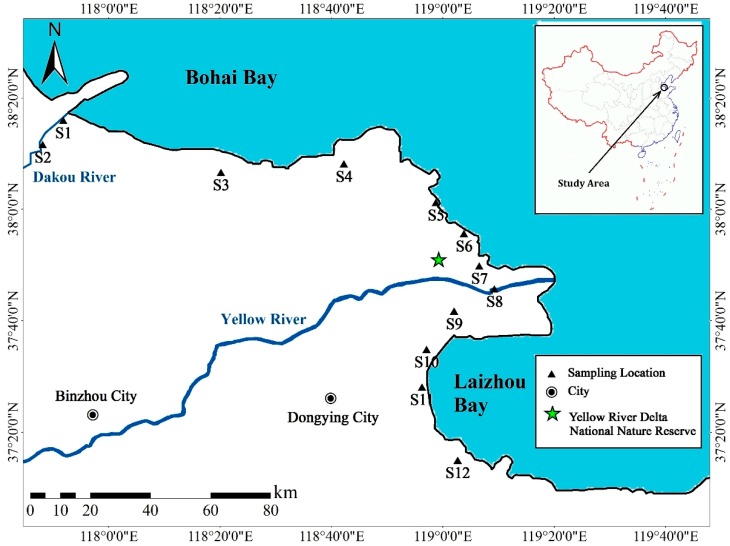
Locations of the sampling sites in the intertidal zones of Yellow River Delta.

The study area is situated on the intertidal zones of the Yellow River delta, China (Figure 1; Table S1 Supporting Information). It is rainy from June to August owing to the temperate continental monsoon climate. The Yellow River and the Dakou River are the two main rivers in the studied area. In addition, some anthropogenic activities such as the discharge of industrial effluents and fish culture existed in the studied area.

In 2014, two sampling events were conducted in April and September. In each event, twelve sediment samples were collected (Figure 1). And ten of them were collected in the intertidal zones along the coastline except S2 and S8, which were separately sampled in the bench land of the Dakou River and the Yellow River for investigating the impacts of human activities. Surface sediment samples were mainly collected using a stainless steel static gravity corer. Approximately the top 5 cm of sediments were removed carefully with a stainless-steel spoon, mixed well and stored in pre-cleaned aluminum containers. The collected sediment samples were air-dried, ground with an agate mortar and then sieved through a 0.149 mm polyethylene sieve. The ground samples were stored in polyethylene Ziploc bags under 4 °C before further use.

### 2.2. Sample Preparation and Analysis

The physicochemical property parameters of sediments were shown in Table S2 (Supporting Information). The pH of sediments was measured with a pH meter (Mettler Toledo FE20, Zurich, Switzerland) at a sediment to water ratio of 1:2.5. Cation exchange capacity (CEC) was determined using compulsive exchange method with BaCl_2_ [19]. The organic matter (OM) in sediments was measured by the combustion method. Sediment particle size distribution was determined by a laser particle size analyzer (Microtrac S3500, Orlando, FL, USA). Sediments (0.1 g) was digested with 3 mL HNO_3_, 1 mL HClO_4_ and 1 mL HF in closed Teflon vessels for 5 h at 165 °C. After cooling, the vessels were transferred to an electric hot plate (160 °C) to eliminate silicon and any remaining HF. After the white smoke disappeared, the samples were taken off and 1 mL HNO_3_ was added and they were then adjusted to 10 mL with ultrapure water to measure the metal(loid)s (As, Cd, Cr, Cu, Mn, Ni, Pb and Zn) by inductively coupled plasma-atomic emission spectrometry (ICP-AES) (Jarrel-ASH ICAP-9000, Franklin, MA, USA). To ensure the analytical quality, geochemical standard soil samples (GSS-1 and GSS-2) provided by the National Research Center for Geoanalysis of China were used to validate the analytical method. The recoveries of standard samples ranged from 89% to 105%.

The sequential extraction of metal(loid)s in sediments was carried out according to the modified European Community Bureau of Reference (BCR) sequential extraction procedure [20]. This procedure separates metal(loid)s in sediments in three fractions, that is, exchangeable and carbonate-associated (F1), reducible (bound to Fe/Mn oxides, F2) and oxidizable (bound to OM, F3) fractions, which are separately extracted using 0.11 M acetic acid solution, 0.1 M hydroxylamine hydrochloride solution adjusted to pH 2.0 with HNO_3_ and 1 M ammonium acetate solution after digestion with H_2_O_2_. To obtain the mass balance of metal(loid)s in sediments, the residual fraction (F4) was measured after acid digestion as procedure above for the total metal determination. The recovery percentage of the sequential extraction was calculated using the following equation:

(1) $$\mathrm{Recovery}\%=\frac{F1+F2+F3+F4}{C_{\mathrm{total}}}\times \%$$

The mean (±SE) recoveries of different elements were 108 ± 2%, 99 ± 4%, 102 ± 5%, 112 ± 1%, 98 ± 2%, 100 ± 3%, 105 ± 3%, 113 ± 5% for As, Cd, Cr, Cu, Mn, Ni, Pb and Zn, respectively, indicating that the BCR sequential extraction worked reasonably well. 

### 2.3. Environmental Assessment

#### 2.3.1. Geo-accumulation Index (*I*_geo_)

The degree of metal enrichment can be assessed using the Geo-accumulation Index (*I*_geo_) introduced by Müller (1969) [21]. The *I*_geo_ calculation is based on the following equation:

(2) $$I_{\mathrm{geo}}=\log_{2}\frac{C_{n}}{1.5\;B_{n}}$$

where *C_n_* is the measured concentration of metal in the sediment, *B_n_* is the geochemical background concentration for the same elements (*n*) and factor 1.5 is the background matrix correction factor due to lithological variations. In this study, the background values of heavy metals and As were used as *B_n_* (As: 9.3; Cd: 0.084; Cr: 66; Cu: 24; Ni: 25.8; Mn: 644; Pb: 25.8; Zn: 63.5 mg kg^−1^) [22]. The *I*_geo_ includes seven grades: uncontaminated (*I*_geo_ ≤ 0), uncontaminated to moderately contaminated (0 < *I*_geo_ < 1), moderately contaminated (1 < *I*_geo_ < 2), moderately to strongly contaminated (2 < *I*_geo_ < 3), strongly contaminated (3 < *I*_geo_ < 4), strongly to extremely contaminated (4 < *I*_geo_ < 5) and extremely contaminated (*I*_geo_ > 5).

#### 2.3.2. Risk Assessment Code (RAC)

The risk assessment code (RAC) is applied to evaluate the environmental risk according to the percentage of F1 fraction for heavy metals in sediments [23,24]. The environmental risks were classified as minimal, low, medium, high and very high if RAC is ≤1%, 1–10%, 10–30%, 30–50% and >50%, respectively.

#### 2.3.3. Potential Ecological Risk Index (PERI)

The potential ecological risk parameter is an effective method for assessing the pollution status of the heavy metal in sediments and its ecological effects, which was initially introduced by Hakanson (1980) [25]. The formulas are as follows:

(3) $$E_{r}^{i}=T_{r}^{i}\cdot C_{fi}$$

(4) $$EI=\sum_{i=0}^{n}E_{r}^{i}$$

where $E_{r}^{i}$ is the potential ecological risk factor of a given metal, *RI* is the comprehensive potential ecological risk index of the metals in the sampling site and $T_{r}^{i}$ is the biological toxicity factor of an individual element, which was determined for Cu = Pb = Ni = 5, Mn = Zn = 1, As = 10, Cr = 2 and Cd = 30. The $E_{r}^{i}$ and RI both contain five grades: low ecological risk ($E_{r}^{i}$ < 40), moderate ecological risk (40 < $E_{r}^{i}$ ≤ 80), appreciable ecological risk (80 < $E_{r}^{i}$ ≤ 160), high ecological risk (160 < $E_{r}^{i}$ ≤ 320) and serious ecological risk ($E_{r}^{i}$ > 320); low ecological risk (*RI* < 150), moderate ecological risk (150 ≤ *RI* < 300), considerable ecological risk (300 ≤ *RI* < 600) and very high ecological risk (*RI* > 600).

#### 2.3.4. Sediment Quality Guidelines (SQGs)

Sediment quality guidelines (SQGs) have been developed for marine ecosystems to provide an interpretive basis for evaluating the risks posed to sediment-dwelling organisms by sediment-associated contaminants [16,26]. In this study, two empirical SQGs which were the effect range-low (ERL)/effect range-median (ERM) and the TEL/PEL were applied to evaluate the potential risk of the ecosystem. In general, the concentrations below the ERL guidelines represent the conditions where biological effects are rarely observed. With metal concentrations falling between those in the ERL and ERM guidelines, it represents a range where biological effects occasionally occur. Concentrations at or above the ERM guidelines represent a probable effect range where adverse biological effects frequently occur. Similarly, metal concentrations that are lower than TEL guidelines represent that negative ecological effects are not expected to occur. Concentrations between those stated in the TEL and PEL guidelines are occasionally correlated to negative ecological effects, while metal concentrations higher than the PEL guidelines represent that negative ecological impacts are frequently to be observed [17].

### 2.4. Statistical Analysis

Statistical analyses were performed with the statistical software package SPSS version 20.0 for Windows (International Business Machines Corporation, New York, NY, USA) including principal component analysis (PCA) and Pearson’s correlation. In the PCA, the principal components were calculated based on the correlation matrix and VARIMAX normalized rotation was used. Pairwise comparisons (paired *t*-test) were introduced to examine the seasonal differences. The results were considered as statistically significant if *p*-value < 0.05.

## 3. Results and Discussion

### 3.1. Temporal-Spatial Variation of Total Metal(loid) Concentrations

**Figure 2:**
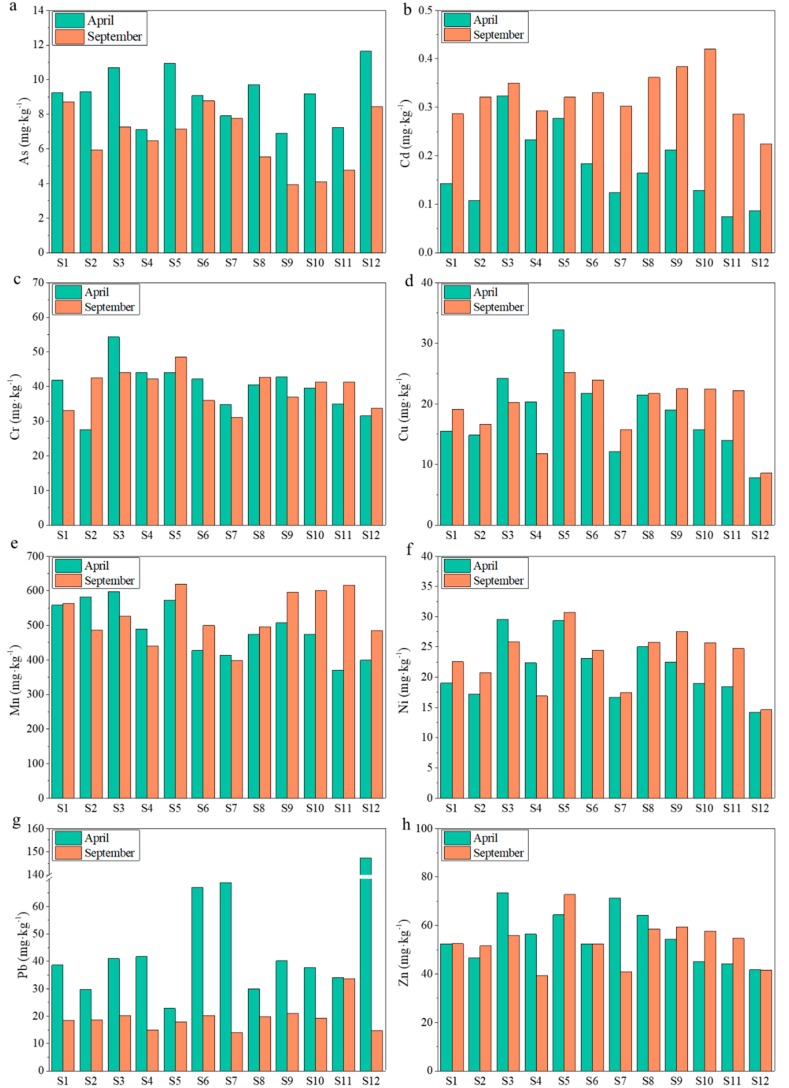
Seasonal and spatial variation of metal(loid)s in surface sediments of intertidal zones. ((**a**): As; (**b**): Cd; (**c**): Cr; (**d**): Cu; (**e**): Mn; (**f**): Ni; (**g**): Pb; (**h**): Zn).

Seasonal and spatial variations of heavy metals and As were shown in Figure 2, which were in the surface sediments of the intertidal zones. In surface sediments, only the concentrations of As, Cd and Pb were significantly different between two seasons (*p* < 0.01). As and Pb presented higher concentrations in April than them in September. Contrarily, the higher concentration of Cd was observed in September instead of in April. The effect of hydrodynamics was the important factor for the seasonal differences of heavy metals or As. And the water level was higher in September than in April. As and Pb showed the conservative behavior and their forms were more stable when the water level was low in the Yellow River estuary [27,28]. Cd was controlled by the transformation mechanisms between the dissolved and particulate state [29]. Moreover, it was attributed to the characteristics of Cd which could be affected easily by some factors such as the concentration of suspended solids and the organic matter content [30]. The average concentration of organic matter in April was lower than that in September (Table S2) and the organic matter could promote the Cd adsorption or complexation [31,32]. No significant seasonal variations were observed for Cr, Cu, Mn, Ni and Zn in surface sediments (*p* > 0.05). The result indicated that most of the heavy metals concentrations were not influenced violently by the changes of short-term environmental conditions between seasons.

All the heavy metals and As exhibited great variations among different sampling sites in both seasons (Figure 2). In order to identify the severity of heavy metal or As contamination in the sediments of the sampling sites, background concentrations of heavy metals and As in the sediments of intertidal zones were compared with them. Based on this, the sources of heavy metals and As could be further pinpointed. In April, the S2, S3, S5, S8 and S12 sediments had relatively higher total As concentrations at 9.30, 10.70, 10.94, 9.71 and 11.64 mg·kg^−1^, respectively, which were 1.00–1.25 times of the background total As concentration. Sewage discharge into the sea may be the reason for the higher concentrations of As in these samples. Total As concentrations in other sites were lower than the background concentration. Total Cd concentrations in the sediments at the sites of S3, S4, S5, S6 and S9 were much higher than the background concentration of 0.084 mg·kg^−1^, which were about 2.00–4.00 times of it. The results were mainly related to the anthropogenic activities such as fish culture, which could lead to the pollution of sediments in these sample sites. A significant positive correlation was found between total Cd concentrations and organic matter contents (*p* < 0.05) (Table S3 Supporting Information), which indicated that the organic matter was an important carrier for Cd. At the sites of S6, S7 and S12, the total Pb concentrations were separately 67.00, 68.77 and 147.34 mg·kg^−1^ which were 2.60, 2.67, 5.71 times of the background concentration. Total Zn concentrations in the sediments at the sites of S3, S5, S7 and S8 were slightly higher than the background concentration. In the sediments of S3 and S5, higher total Cu concentrations were observed at 24.20 and 32.21 mg·kg^−1^, respectively. Accordingly, the both sites also had higher total Ni concentrations of 29.52 and 29.33 mg·kg^−1^, respectively, implying that Cu and Ni were likely released from the same sources. They both had significant positive correlations with the organic matter contents (Table S3). Total Cr and Mn concentrations at the sampling sites were all lower than their background concentrations.

In September, total Cd concentrations at all the sites were 2.50–5.00 times higher than its background concentration. Conversely, total As, Cr and Mn concentrations at the sites were all lower than their background concentrations. Total Cu and Zn concentrations had the highest value at the site of S5 and total Pb concentrations had the highest value at S11. The three heavy metals concentrations at other sites were all lower than the background concentrations. Total Ni concentrations in the sediments at the sites of S3, S5 and S9 were slightly higher than the background concentration. Therefore, the concentration characteristics of heavy metals and As indicated a minimal contamination by external sources except Cd in September.

In addition to the effect of hydrodynamics for the seasonal differences, the sediment grain size (Table S2) was also the important factor for the distribution of heavy metals. The heavy metal contents can gradually increase along with the change of the sediment particle-size from coarse to fine. It was attributed to the greater surface areas and adsorption exchange capacities for the fine particles in sediments [33,34]. What’s more, seasonal variation can also cause the change of the biological activity including plants, animals, or microorganisms, which play a very important role in the accumulation and migration of heavy metals and As [35,36].

**Figure 3:**
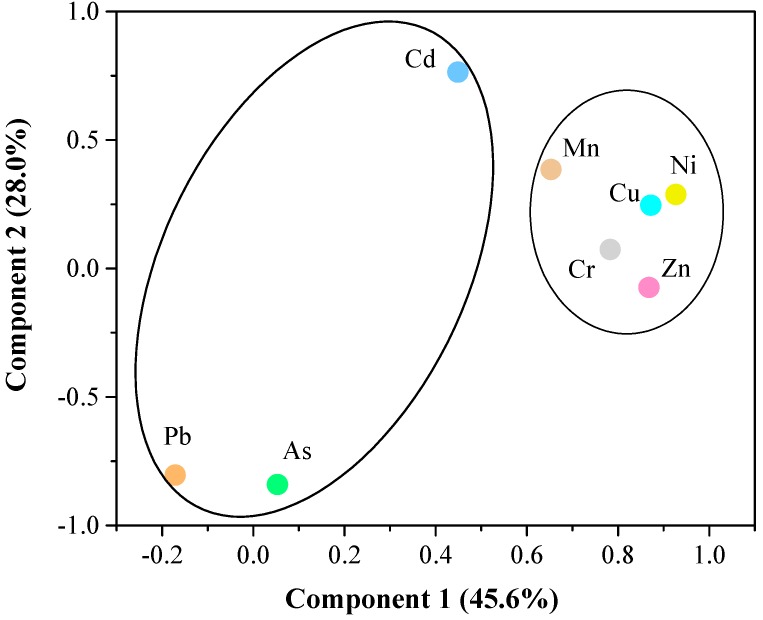
Principal component analysis of total metal(loid) concentrations in sediments.

The PCA (Figure 3 and Table 1) showed that Cr, Cu, Mn, Ni and Zn were clustered to one component (PC 1) accounting for 45.59% of the variance and As, Pb and Cd to the second component (PC 2) accounting for 28.00% of the variance. To sum up, the two components explained 73.59% of the variance.

Different heavy metals and As were grouped into PC 1 or 2, indicating that they might be derived from common sources. Cr and Ni belong to the siderophile elements, which are the primary rock-forming elements [37]. It is easy for them to integrate with iron magnesium silicate minerals due to their similar ionic radius [38]. They are derived from terrigenous detritus material transported by surface runoff [39], indicating a natural input. Therefore, the heavy metals (grouped into PC 1) might be mainly from natural sources. In addition, As, Pb and Cd were grouped into PC 2, indicating that they might originate from another common source. The pollution in the coastal water and the oil exploitation in this region might be important sources for them. Moreover, Tang et al. (2010) observed that higher As and Cd contents in the seawater of the Yellow River Estuary were primarily affected by inputs from the Yellow River [40].

### 3.2. Temporal-Spatial Variation of the Metal(loid) Speciation

**Figure 4:**
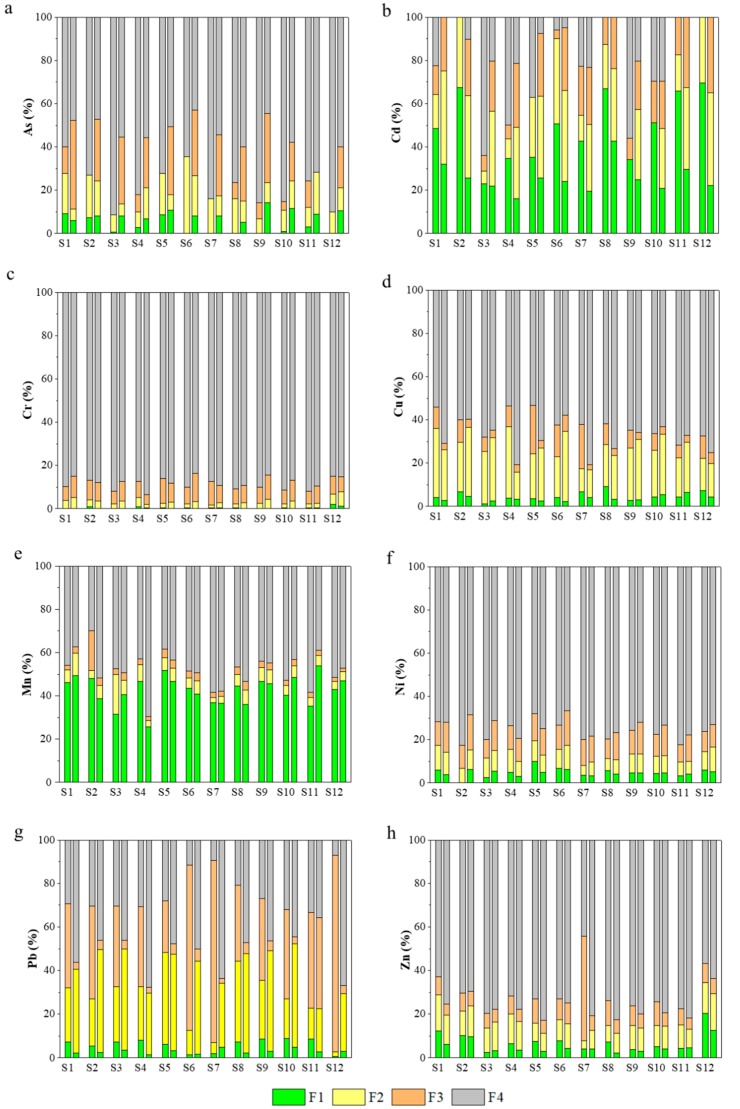
Fractions of bioavailable and carbonate-associated (F1), reducible (F2), oxidizable (F3) and residual (F4) heavy metals and As in sediments (the columns corresponding to each sampling site: the left column represented the percentages of four fractions in April; the right column represented the percentages of four fractions in September). ((**a**): As; (**b**): Cd; (**c**): Cr; (**d**): Cu; (**e**): Mn; (**f**): Ni; (**g**): Pb; (**h**): Zn).

The percentages of four heavy metal fractions in sediments (i.e., F1–F4) as previously described are shown in Figure 4. The exchangeable and carbonate-associated (F1), reducible (bound to Fe/Mn oxides, F2) and oxidizable (bound to OM, F3) fractions are extractable and have the biological effectiveness [41]. There exist some potential hazards for the three forms. Furthermore, the anthropogenic pollution has a great impact on these forms [42]. Nevertheless, the residual fraction (F4) with minimal toxicity is very stable, which is difficult to migrate or transform under general conditions [43,44].

In April, the F4 fraction was predominant and the exchangeable and the F1 fraction was relatively lower for As, Cr, Cu and Ni in the sediments. The F4 fraction was also dominant for Zn in most sampling sites. Because Zn mainly came from the natural weathering of rock minerals, thus making the proportion of the F4 fraction relatively high. The ranges of the F1 and F4 fractions for the five metal(loid)s were separately 0.52–7.68%, 62.12–89.06% for all the sampling sites. Because the F4 fraction is imbedded in the silicate crystalline structures of sediments, this fraction of heavy metals is very stable and unlikely to be released to pore water [5]. The results were also supported by the Pearson’s correlation analysis (Table 2), in which a very significant positive correlation was found between the five metal(loid)s and the F4 fraction (*p* < 0.01). Therefore, these results indicated that the study area had a lower environmental risk of As, Cr, Cu, Ni and Zn. In the sediments of most sampling sites, the F3 fraction was the main form of Pb averaging 48.76%, whereas the corresponding F1 fractions were also very low with a mean value of 5.97%. The results indicated the organic matter had a stronger geochemical affinity to Pb, resulting in higher F3 fraction of Pb. The fraction of Pb can be released to pore water under potential environmental risks [5,23].

However, the F1 fraction of Cd was dominant in the sediments for most sampling sites averaging 49.14%. What’s more, the sediments of total Cd concentrations such as S2, S8, S10, S11 and S12 had relatively higher F1 percentage at 67.41%, 66.84%, 51.17%, 65.89% and 69.62%, respectively, implying that external sources more likely contributed to the more bioavailable and mobile forms of Cd. The F1 and F4 fractions were the dominant forms for Mn averaging 42.83% and 47.07%, respectively. Meanwhile, total Mn concentration and its F1 fraction presented a significant positive correlation (*p* < 0.01). The results indicated that Mn in the sediments had the higher biological effectiveness. And it is easy to migrate or transform.

In September, the F4 fraction was predominant and the F1 fraction was very low for most heavy metals and As except Cd and Mn in the sediments. The significant positive correlations were also found between total As, Cr, Cu, Ni, Pb or Zn concentration and the corresponding F4 fraction (Table 2). In general, the results indicated that the study area had a lower environmental risk of As, Cr, Cu, Ni, Pb and Zn in September. The dominant forms for Mn were the F1 and F4 fractions averaging 42.46% and 48.85%, respectively. And the significant positive correlations between total Mn concentration and its F1 fraction (*p* < 0.01) and a negative correlation between total Mn concentration and its F4 fraction were observed (Table 2). But with respect to Cd, the main form was the F2 fraction averaging 36.10%, followed by the F3 fraction and the F1 fraction with the mean value of 27.00% and 25.44%, respectively. The extractable fractions take up a large proportion for Cd, indicating a high environmental risk.

### 3.3. Environmental Risk Assessment

In April, the *I*_geo_ values of As, Cr, Cu, Mn, Ni and Zn at all sites, Cd at the site 2, 7, 11, 12 and Pb at the site 1, 2, 5, 8, 10, 11 were less than or at zero (Table 3), suggesting that these sites were not polluted. The values of *I*_geo_ for Cd at the site 1, 4, 6, 8, 9, 10 and Pb at the site 3, 4, 6, 7, 9 were from 0 to 1 in the sediments which usually had “unpolluted to moderately polluted” class, while the *I*_geo_ values of Cd at the site 3, 5 and Pb at the site 12 were both from 1 to 2, suggesting that the *I*_geo_ class in the sediments of these sites was “moderately contaminated”. The results showed that the accumulation of Cd and Pb in the season was relatively serious in surface sediments of some sites.

Sediments of some sampling sites were heavily polluted by Cd, of which the higher environmental risk was found due to higher percentages of the bioavailable/mobile fraction (i.e., the F1 fraction) (Figure 4, Table 4). And sediments of most sampling sites presented high environmental risks induced by Mn (Table 4). Although S12 showed heavy Pb pollution according to the *I*_geo_ values, no environmental risk was induced by Pb in this site (Table 4). On the contrary, Zn showing relatively lower pollution levels had a medium environmental risk for S1, S2 and S12 (Table 3 and Table 4). As, Cr, Cu and Ni had no or low environmental risks for the studied sediments.

The results for metal(loid) concentration in the surface sediment according to the potential ecological risk index (PERI) were shown in Table 5. In April, the $E_{r}^{i}$ values of As, Cr, Cu, Mn, Ni, Pb and Zn were all less than 40, which showed low ecological risk. Also, the values of *RI* at all sites were less than 150, which indicated low ecological risk. But the $E_{r}^{i}$ value of Cd was from 40 to 80, indicating moderate ecological risk.

The concentration of heavy metals and As in the sediment samples were contrasted with the consensus-based ERL, ERM, TEL and PEL values (Table 6). In April, the results show that As, Ni and Pb were between ERL and ERM for 66.67%, 50% and 25% and were between TEL and PEL for 83.33%, 91.67%, 66.67% of the samples, respectively, indicating that the concentration of As, Ni or Pb was likely to occasionally exhibit adverse effects on the ecosystem. However, the concentrations of Cd were all below the ERL and TEL guidelines showing that the biological effects were rarely observed and negative ecological effects were not expected to occur.

In September, the *I*_geo_ values of As, Cr, Cu, Mn, Ni, Pb and Zn at all sites were less than zero (Table 3), suggesting that these sites were not polluted. The value of *I*_geo_ for Cd at the site 12 were from 0 to 1 which had “unpolluted to moderately polluted” class but the *I*_geo_ values of Cd at all other sites were from 1 to 2, suggesting that the *I*_geo_ class was “moderately contaminated.” The results showed that the accumulation of Cd in the season was relatively serious in surface sediments.

Some sediments were also heavily polluted by Cd in September, which also had the higher environmental risk because of the higher percentages of the F1 fraction (Figure 4, Table 4). And sediments of most sampling sites also presented high environmental risks induced by Mn (Table 4). As had a medium environmental risk for the sediments of S5, S9, S10 and S12, which were different from the results in April. Only S12 presented a medium environmental risk induced by Zn and other sediments showed lower pollution levels. Cr, Cu, Ni and Pb had no or low environmental risks for the studied sediments.

The $E_{r}^{i}$ values of As, Cr, Cu, Mn, Ni, Pb and Zn were all less than 40, which showed low ecological risk. The $E_{r}^{i}$ value of Cd was from 80 to 160, indicating appreciable ecological risk in September. The values of *RI* at the sites 8, 9 and 10 were from 150 to 300, which indicated moderate ecological risk. Site 8 was located at the entrance of the Yellow River which flowed through many densely populated areas and received a large amount of wastewater. Besides, there were some irrigated or aquiculture regions around which could lead to the serious pollution in the sites 9 and 10.

The results show that As and Ni were between ERL and ERM for 25%, 66.67% and between TEL and PEL for 41.67% and 91.67% of the samples (Table 6), respectively, indicating that the concentration of As or Ni was likely to occasionally exhibit adverse effects on the ecosystem in September. However, the concentrations of Cd were also all below the ERL and TEL guidelines showing that adverse effects on the ecosystem were rarely observed.

## 4. Conclusions

All the heavy metals and As exhibited great variations among different sampling sites in both seasons. Only the concentrations of As, Cd and Pb were significantly different between April and September (*p* < 0.01). In April, the F4 fraction was predominant for As, Cr, Cu, Ni and Zn in the sediments. But the F1 fraction of Cd was dominant in the sediments for most sampling sites averaging 49.14%. In September, the F4 fraction was predominant and the F1 fraction was very low for most metal(loid)s except Cd and Mn in the sediments. The main form of Cd was the F2 fraction, averaging 36.10% and the proportion of factions having the biological effectiveness was 88.54%. The *I*_geo_, the F1 fraction and PERI of most heavy metals were relatively low in the surface sediments for both seasons. But Pb, As and Ni were between TEL and PEL for 66.67%, 83.33% and 91.67% in April and As and Ni were between TEL and PEL for 41.67% and 91.67%, which indicated that the concentration of them was likely to occasionally exhibit adverse effects on the ecosystem. Although the *I*_geo_, the F1 fraction or PERI of Cd in both seasons was higher in surface sediments at some sites, the results of sediment quality guidelines (SQGs) showed that the biological effects were rarely observed and negative ecological effects were not expected to occur in the studied area.

## Figures and Tables

**Table 1:** Total variance explained and rotated component matrix for total concentrations of metal(loid)s in the sediments.

Component	Initial Eigenvalues			Rotation Sums of Squared Loadings		
	Total	% of Variance	Cumulative %	Total	% of Variance	Cumulative %
1	4.302	53.778	53.778	3.647	45.590	45.590
2	1.585	19.809	73.587	2.240	27.997	73.587
3	0.630	7.870	81.457			
4	0.507	6.339	87.796			
5	0.371	4.640	92.435			
6	0.354	4.422	96.857			
7	0.219	2.741	99.598			
8	0.032	0.402	100.000			

Extraction method: principal component analysis; rotation method: VRAIMAX with Kaiser normalization.

**Table 2:** Pearson’s correlation between total concentration and speciation of heavy metals or As in the sediments in April and September (*n* = 12).

Month	Speciation	As	Cd	Cr	Cu	Mn	Ni	Pb	Zn
April	F1	0.230	0.527	−0.466	0.293	0.797 **	0.649 *	−0.524	−0.332
	F2	0.388	0.282	0.235	0.842 **	0.636 *	0.794 **	−0.619 *	0.051
	F3	−0.453	0.124	0.403	0.774 **	0.481	0.848 **	0.993 **	0.546
	F4	0.818 **	0.893 **	0.992 **	0.951 **	0.236	0.958 **	−0.002	0.716 **
September	F1	0.593 *	0.529	−0.249	0.439	0.935 **	0.795 **	0.540	−0.214
	F2	0.370	0.516	−0.184	0.885 **	0.666 *	0.658 *	0.351	0.216
	F3	0.804 **	0.397	0.359	0.631 *	0.682 *	0.898 **	0.909 **	0.633 *
	F4	0.853 **	0.658 *	0.983 **	0.929 **	−0.038	0.969 **	0.673 *	0.968 **

* Correlation is significant at the 0.05 level (2-tailed); ** Correlation is significant at the 0.01 level (2-tailed).

**Table 3:** The values of Geo-accumulation indices (*I*_geo_) of heavy metals and As for sediments of all sites in April and September.

Month	Sites	*I*_geo_							
		As	Cd	Cr	Cu	Mn	Ni	Pb	Zn
April	S1	−0.59	0.19	−1.24	−1.22	−0.79	−1.02	0.00	−0.86
	S2	−0.58	−0.23	−1.84	−1.28	−0.73	−1.17	−0.38	−1.03
	S3	−0.38	1.36	−0.87	−0.57	−0.69	−0.39	0.08	−0.37
	S4	−0.97	0.89	−1.17	−0.82	−0.98	−0.79	0.11	−0.75
	S5	−0.35	1.14	−1.17	−0.16	−0.76	−0.40	−0.76	−0.56
	S6	−0.62	0.54	−1.23	−0.73	−1.18	−0.75	0.79	−0.86
	S7	−0.82	−0.03	−1.51	−1.57	−1.22	−1.22	0.83	−0.42
	S8	−0.52	0.38	−1.29	−0.74	−1.03	−0.63	−0.37	−0.57
	S9	−1.01	0.75	−1.21	−0.92	−0.93	−0.78	0.05	−0.81
	S10	−0.60	0.03	−1.33	−1.20	−1.03	−1.03	−0.04	−1.08
	S11	−0.95	−0.76	−1.50	−1.37	−1.38	−1.07	−0.19	−1.11
	S12	−0.26	−0.54	−1.65	−2.20	−1.28	−1.45	1.93	−1.19
September	S1	−0.68	1.19	−1.58	−0.92	−0.78	−0.78	−1.07	−0.86
	S2	−1.23	1.35	−1.22	−1.11	−0.99	−0.90	−1.05	−0.88
	S3	−0.94	1.47	−1.17	−0.83	−0.88	−0.58	−0.94	−0.77
	S4	−1.11	1.22	−1.23	−1.61	−1.14	−1.20	−1.38	−1.27
	S5	−0.97	1.35	−1.03	−0.52	−0.64	−0.33	−1.11	−0.39
	S6	−0.67	1.39	−1.46	−0.59	−0.95	−0.66	−0.94	−0.87
	S7	−0.85	1.26	−1.67	−1.19	−1.28	−1.15	−1.48	−1.22
	S8	−1.33	1.52	−1.21	−0.73	−0.96	−0.59	−0.96	−0.70
	S9	−1.82	1.61	−1.42	−0.68	−0.70	−0.49	−0.89	−0.68
	S10	−1.77	1.74	−1.26	−0.68	−0.69	−0.59	−1.01	−0.73
	S11	−1.54	1.18	−1.26	−0.70	−0.65	−0.64	−0.20	−0.80
	S12	−0.72	0.83	−1.55	−2.07	−0.99	−1.41	−1.39	−1.20

**Table 4:** Metal risk assessment codes (RACs) for sediments of all sites in April and September.

Month	Sites	RAC							
		As	Cd	Cr	Cu	Mn	Ni	Pb	Zn
April	S1	L	H	N	L	H	L	L	M
	S2	L	VH	N	L	H	N	L	M
	S3	N	M	N	L	H	L	L	L
	S4	L	H	N	L	H	L	L	L
	S5	L	H	N	L	VH	L	L	L
	S6	N	VH	N	L	H	L	L	L
	S7	N	H	N	L	H	L	L	L
	S8	N	VH	N	L	H	L	L	L
	S9	N	H	N	L	H	L	L	L
	S10	N	VH	N	L	H	L	L	L
	S11	L	VH	N	L	H	L	L	L
	S12	N	VH	L	L	H	L	N	M
September	S1	L	H	N	L	H	L	L	L
	S2	L	M	N	L	H	L	L	L
	S3	L	M	N	L	H	L	L	L
	S4	L	M	N	L	M	L	L	L
	S5	M	M	N	L	H	L	L	L
	S6	L	M	N	L	H	L	L	L
	S7	L	M	N	L	H	L	L	L
	S8	L	H	N	L	H	L	L	L
	S9	M	M	N	L	H	L	L	L
	S10	M	M	N	L	H	L	L	L
	S11	L	M	N	L	VH	L	L	L
	S12	M	M	L	L	H	L	L	M

*N*: no risk; *L*: low risk; *M*: medium risk; *H*: high risk; *VH*: very high risk.

**Table 5:** Potential ecological risk indices $E_{r}^{i}$ and RI for studied metals in April and September.

Month	Sites	$E_{r}^{i}$								RI	Risk Grade
		As	Cd	Cr	Cu	Mn	Ni	Pb	Zn		
April	S1	9.94	51.16	1.27	3.23	0.87	3.69	7.49	0.82	78.48	Low
	S2	10.00	38.35	0.84	3.09	0.90	3.33	5.75	0.73	63.00	Low
	S3	11.51	115.43	1.65	5.04	0.93	5.72	7.95	1.16	149.38	Low
	S4	7.66	83.32	1.33	4.24	0.76	4.33	8.09	0.89	110.62	Low
	S5	11.76	98.93	1.34	6.71	0.89	5.68	4.42	1.01	130.75	Low
	S6	9.76	65.63	1.28	4.52	0.66	4.47	12.98	0.82	100.13	Low
	S7	8.50	44.16	1.05	2.53	0.64	3.23	13.33	1.12	74.56	Low
	S8	10.44	58.76	1.23	4.48	0.74	4.85	5.82	1.01	87.32	Low
	S9	7.42	75.78	1.30	3.96	0.79	4.36	7.79	0.85	102.25	Low
	S10	9.88	45.93	1.20	3.27	0.74	3.67	7.30	0.71	72.69	Low
	S11	7.78	26.49	1.06	2.91	0.57	3.57	6.59	0.70	49.66	Low
	S12	12.51	30.86	0.95	1.63	0.62	2.75	28.55	0.66	78.53	Low
	Mean	9.76	61.23	1.21	3.80	0.76	4.14	9.67	0.87		
	$E_{r}^{i}$ grade	Low	Moderate	Low	Low	Low	Low	Low	Low		
September	S1	9.39	102.38	1.00	3.98	0.88	4.37	3.56	0.83	126.38	Low
	S2	6.37	114.85	1.29	3.46	0.75	4.01	3.62	0.81	135.17	Low
	S3	7.81	125.00	1.33	4.21	0.82	5.00	3.92	0.88	148.98	Low
	S4	6.94	104.58	1.28	2.46	0.68	3.27	2.88	0.62	122.73	Low
	S5	7.67	114.64	1.47	5.24	0.96	5.95	3.47	1.15	140.54	Low
	S6	9.43	117.95	1.09	4.98	0.78	4.73	3.92	0.82	143.70	Low
	S7	8.33	107.98	0.94	3.29	0.62	3.38	2.69	0.65	127.88	Low
	S8	5.97	129.19	1.29	4.52	0.77	4.99	3.85	0.92	151.50	Moderate
	S9	4.24	136.94	1.12	4.69	0.93	5.33	4.06	0.94	158.24	Moderate
	S10	4.40	149.91	1.25	4.68	0.93	4.98	3.72	0.91	170.78	Moderate
	S11	5.14	102.29	1.25	4.62	0.96	4.80	6.52	0.86	126.43	Low
	S12	9.09	80.07	1.02	1.78	0.75	2.83	2.86	0.65	99.06	Low
	Mean	7.07	115.48	1.20	3.99	0.82	4.47	3.75	0.84		
	$E_{r}^{i}$ grade	Low	Appreciable	Low	Low	Low	Low	Low	Low		

*Low* means low ecological risk, *Moderate* means moderate ecological risk and *Appreciable* means appreciable ecological risk.

**Table 6:** Comparison between the ERL/ERM or TEL/PEL value and the heavy metal or As concentration of all samples in April and September.

Month		As	Cd	Cr	Cu	Mn	Ni	Pb	Zn
April	ERL^a^ (mg·kg^−1^)	8.2	1.2	81	34	-	20.9	46.7	150
	ERM^a^ (mg·kg^−1^)	70	9.6	370	270	-	51.6	218	410
	TEL^b^ (mg·kg^−1^)	7.24	0.68	52.3	18.7	-	15.9	30.2	124
	PEL^b^ (mg·kg^−1^)	41.6	4.21	160	108	-	42.8	112	271
	<ERL (%)	33.33	100	100	100	-	50	75	100
	ERL-ERM (%)	66.67	0	0	0	-	50	25	0
	>ERM (%)	0	0	0	0	-	0	0	0
	<TEL (%)	16.67	100	91.67	50	-	8.33	25	100
	TEL-PEL (%)	83.33	0	8.33	50	-	91.67	66.67	0
	>PEL (%)	0	0	0	0	-	0	8.33	0
September	<ERL (%)	75	100	100	100	-	33.33	100	100
	ERL-ERM (%)	25	0	0	0	-	66.67	0	0
	>ERM (%)	0	0	0	0	-	0	0	0
	<TEL (%)	58.33	100	100	33.33	-	8.33	91.67	100
	TEL-PEL (%)	41.67	0	0	66.67	-	91.67	8.33	0
	>PEL (%)	0	0	0	0	-	0	0	0

^a^ ERL and ERM guidelines quoted from Long et al. (1995); ^b^ TEL and PEL guidelines quoted from Macdonald et al. (1996) [45].

## Supplementary Materials

The following are available online at http://www.mdpi.com/1660-4601/15/10/2125/s1, Table S1: Descriptions of the sampling sites, Table S2: Physicochemical properties of surface sediments in both seasons, Table S3: Pearson’s correlation between total heavy metal concentrations and OM contents in the sediments in April and September (*n* = 12).
