# Supplementary material for: Speciation Variation and Comprehensive Risk Assessment of Metal(loid)s in Surface Sediments of Intertidal Zones
Source: Int J Environ Res Public Health. 2018 Sep 27;15(10):2125. doi: 10.3390/ijerph15102125 (PMC6211063; doi:10.3390/ijerph15102125)
Supplement: Supplementary file 1 [file ijerph-15-02125-s001.pdf]

International Journal of
*Environmental Research and Public Health*

MDPI

*Supplementary Materials*

# Speciation Variation and Comprehensive Risk Assessment of Metal(loid)s in Surface Sediments of Intertidal Zones

**Baocui Liang, Xiao Qian, Shitao Peng, Xinhui Liu *, Lili Bai, Baoshan Cui and Junhong Bai**

**Table S1.** Descriptions of the sampling sites.

| Site | Location | Surronding Land Type |
|---|---|---|
| S1 | N38°15'59.52", E117°51'45.65" | fish farms |
| S2 | N38°11'38.05", E117°48'4.34" | estuary |
| S3 | N38° 6'39.32", E118°20'6.49" | fish farms |
| S4 | N38° 8'11.11", E118°42'10.93" | fish farms |
| S5 | N38° 1'15.01", E118°58'42.64" | Harbou |
| S6 | N37°55'41.27", E119° 3'45.46" | Oilfield |
| S7 | N37°49'11.57", E119° 6'30.97" | nature reserve |
| S8 | N37°45'43.89", E119° 9'15.36" | estuary |
| S9 | N37°41'44.70", E119° 2'0.42" | fish farms |
| S10 | N37°34'52.99", E118°57'3.22" | Land reclamation |
| S11 | N37°28'6.99", E118°56'13.06" | Industrial park |
| S12 | N37°14'59.96", E119° 2'37.91" | fish farms |

**Table S2.** Physicochemical properties of surface sediments in both seasons.

| Time | Sites | pH | CEC[a)] | OM[b)] | Size distribution (%) | | |
|---|---|---|---|---|---|---|---|
| | | | ($cmo{\cdot}kg^{-1}$) | ($g{\cdot}kg^{-1}$) | Clay[c)] | Silt[d)] | Sand[e)] |
| April | S1 | 8.25 | 6.10 | 38.41 | 21.02 | 71.96 | 7.02 |
| | S2 | 8.09 | 6.51 | 42.36 | 19.61 | 74.75 | 5.64 |
| | S3 | 8.16 | 8.77 | 47.08 | 30.77 | 68.17 | 1.06 |
| | S4 | 8.17 | 8.69 | 26.37 | 0.00 | 55.27 | 44.73 |
| | S5 | 7.87 | 10.11 | 85.52 | 26.13 | 71.04 | 2.83 |
| | S6 | 8.02 | 5.44 | 32.82 | 7.06 | 87.51 | 5.43 |
| | S7 | 8.46 | 3.91 | 32.84 | 0.00 | 50.89 | 49.11 |
| | S8 | 8.59 | 7.49 | 28.69 | 12.83 | 85.30 | 1.87 |
| | S9 | 7.93 | 8.05 | 46.87 | 23.72 | 75.24 | 1.04 |
| | S10 | 7.86 | 5.78 | 30.68 | 13.76 | 84.32 | 1.92 |
| | S11 | 8.06 | 4.89 | 23.69 | 0.00 | 90.19 | 9.81 |
| | S12 | 7.88 | 2.91 | 18.90 | 0.00 | 4.37 | 95.63 |
| September | S1 | 8.53 | 11.03 | 46.62 | 2.58 | 71.42 | 26.00 |
| | S2 | 7.89 | 8.56 | 37.94 | 0.71 | 78.10 | 21.19 |
| | S3 | 9.41 | 11.70 | 47.00 | 2.35 | 83.00 | 14.65 |
| | S4 | 7.88 | 11.80 | 55.23 | 1.31 | 74.89 | 23.80 |
| | S5 | 8.22 | 9.86 | 41.81 | 0.00 | 63.89 | 36.11 |
| | S6 | 8.47 | 12.50 | 52.79 | 1.56 | 92.30 | 6.14 |
| | S7 | 8.58 | 5.42 | 28.02 | 0.00 | 36.83 | 63.17 |

| | | | | | | |
|---|---|---|---|---|---|---|
| S8 | 8.54 | 9.19 | 29.10 | 0.38 | 68.95 | 30.67 |
| S9 | 8.57 | 13.80 | 53.76 | 1.75 | 90.72 | 7.53 |
| S10 | 8.05 | 10.32 | 67.18 | 13.76 | 84.32 | 1.92 |
| S11 | 8.11 | 9.94 | 48.05 | 0.00 | 68.52 | 31.48 |
| S12 | 8.11 | 4.12 | 26.39 | 0.00 | 8.76 | 91.24 |

[a)] CEC means cation exchange capacity; [b)] OM means the organic matter; [c)]the range of clay particle size was<3.9 μm; [d)] the particle size of silt ranged from 3.9 to 62.5 μm; [e)] the particle size of sand ranged from 62.5 to 2000 μm.

**Table S3.** Pearson's correlation between total heavy metal concentrations and OM contents in the sediments in April and September (n=12).

| | Month | As | Cd | Cr | Cu | Mn | Ni | Pb | Zn |
|---|---|---|---|---|---|---|---|---|---|
| OM | April | 0.285 | 0.630* | 0.373 | 0.774** | 0.675* | 0.656* | -0.465 | 0.428 |
| | September | -0.417 | 0.556 | 0.239 | 0.418 | 0.500 | 0.393 | 0.282 | 0.234 |

* Correlation is significant at the 0.05 level (2-tailed); ** Correlation is significant at the 0.01 level (2-tailed).

© 2018 by the authors. Submitted for possible open access publication under the terms and conditions of the Creative Commons Attribution (CC BY) license (http://creativecommons.org/licenses/by/4.0/).
